# Aetiology, antimicrobial susceptibility and outcome of children with sepsis, admitted at Muhimbili National Hospital, Dar es Salaam

**DOI:** 10.11604/pamj.2022.42.167.29969

**Published:** 2022-07-01

**Authors:** Evance Godfrey, Edna Majaliwa, Evelyne Neema Assenga

**Affiliations:** 1 Department of Paediatrics and Child Health, Muhimbili National Hospital (MNH), Dar es Salaam, Tanzania,; 2 Department of Paediatrics and Child Health, Muhimbili University of Health and Allied Sciences (MUHAS), Dar es Salaam, Tanzania

**Keywords:** Bacteria sepsis, antimicrobial susceptibity, outcome

## Abstract

**Introduction:**

sepsis is defined as a systemic inflammatory host response syndrome (SIRS) to infection, commonly bacterial. The global prevalence of sepsis is 8.2% with a mortality rate of 25%, whilst in Tanzania the prevalence is 6.6%. Treatment of sepsis involves early initiation of antibiotics based on local sensitivity patterns. However, there is an increase in antimicrobial resistance to commonly used antibiotics. Hence to promote rational use of antibiotics, we aimed at establishing the etiology, local susceptibility patterns and outcome of children with sepsis aged 2 months to 15 years, admitted at Muhimbili National Hospital (MNH), Dar es Salaam.

**Methods:**

a hospital based prospective cross sectional study was conducted among 245 participants who were consecutively recruited. A standardized structured questionnaire was used to collect information. Blood cultures and complete blood counts were done. Antimicrobial susceptibility was also done on positive cultures using disc diffusion method. Data were analyzed using SPSS version 20. Frequencies and proportions were used to summarize categorical data, whilst median and interquartile range was used to summarize continuous data. Student T test was used to compare means of data which were normally distributed and the differences in proportions were tested using Chi square test or Fisher’s exact test. A p value of = 0.05 was considered to be statistically significant.

**Results:**

there was predominance of male participants (67.5%) with a median age was 2 years and an interquartile range (IQR) 10 months to 4 years. Culture positive sepsis was detected among 29.8% of the participants, and the common Gram-positive bacterial isolates were S. aureus (39.7%) Coagulase Negative Staphylococcus (CoNS) (35.6%) and Gram-negative isolates were E. coli (12.3%), Klebsiella spp (6.8%) and Pseudomonas aeruginosa (5.5%). All bacteria showed a high resistance to ampicillin (80%- 100%) followed by ceftriaxone (40 - 70%). All Pseudomonas aeruginosawere 100% resistant to ampicillin, gentamycin and ceftriaxone but were sensitive to amikacin. There was less than 40% resistance to co-amoxiclav, meropenem, ciprofloxacin, amikacin, and clindamycin. The overall case mortality rate from sepsis was 9.4%. Among children discharged 59.3% had prolonged hospital stay of more than 7 days. Age group 1 to 5 years, prior use of antibiotics, tachycardia, and leukocytosis were significantly associated with high mortality.

**Conclusion:**

bacterial sepsis is prevalent at Muhimbili National Hospital contributing to 9.4% of mortality and a prolonged hospital stay of more than 7 days among 59.3% of the participants. Gram-positive bacteria were found to be predominant cause of sepsis, whereas both Gram-positive and Gram-negative bacteria had a high resistance to first and second line antimicrobials including: ampicillin, gentamycin, and ceftriaxone.

## Introduction

Globally, more than 5.8 million children die annually, 1.7 million occur in Asia, 2.4 million in Western and Eastern sub-Saharan Africa. Sepsis and sepsis related deaths account for more than 50% of these deaths [1]. The global prevalence of sepsis was found to be 8.2% with a 3 times higher prevalence in Africa (23.1%), though the mortality rate of 25% was similar in developed and developing countries [2].

The burden is even higher in Tanzania, where every year 154,000 children die before reaching their 5^th^ birthday and 71% of these are due to sepsis-related deaths [3]. Tanzania which is among the African countries, reported a prevalence of 6.6% in 2013 [4]. Therefore, efforts must be made to reduce sepsis-related mortality which contributes to about 50% of all under-five deaths [5]. Bacteria causing sepsis vary between developed and developing countries with some similarities [6-9]. Treatment of sepsis involves early recognition and empirical treatment according to local epidemiology and antimicrobial susceptibility patterns [10]. According to the World Health Organization (WHO), there is an increase in antimicrobial resistance to commonly used antimicrobials for the treatment of sepsis [11]. Similarly, several studies done in our setting among neonates showed an increase in antimicrobial resistance to first-line antimicrobials [11-13]. However, there is limited data on bacteria aetiology and antimicrobial susceptibility pattern in children aged 2 months to 15 years. We therefore aimed at determining the bacterial aetiology, antimicrobial susceptibility, and immediate outcome of these children with sepsis aged 2 months to 15 years, admitted at Muhimbili National Hospital.

The broad objectives were to determine bacterial aetiology, antimicrobial susceptibility, and immediate outcome of these children with sepsis admitted at Muhimbili National Hospital. Specific objectives: 1) to determine commonly bacteria isolated in children with sepsis admitted at Muhimbili National Hospital (MNH); 2) to determine the antimicrobial susceptibility of the common bacteria causing sepsis in children admitted at MNH; 3) to describe the immediate outcome of children with sepsis admitted at MNH.

## Methods

**Study design:** a descriptive hospital-based cross-sectional with a longitudinal follow-up study.

**Setting:** Muhimbili National Hospital is the national referral and university teaching hospital with a 1500 beds capacity, attending 1,000 to 1200 outpatients per day and approximately 1200 admissions per week. The paediatric ward has a bed capacity of 127 with approximately 20 admissions per day. The study was conducted over a 7-month period from September 2018 to March 2019.

**Participants:** children aged 2 months to 15 years admitted to the paediatrics wards were recruited consecutively. We recruited all children who met Systemic Inflammatory Response Syndrome (SIRS) criteria for sepsis. We also included all children who were admitted due to severe acute malnutrition (SAM) as they are presumed to have sepsis, and the criteria for admission for children with SAM was based on SAM and critically ill and excluded all children with malignancies as they can have fever and tachycardia and leukocytosis as the natural history of the disease and not having bacteria sepsis.

**Sample size:** sample size were calculated using


$$n=\frac{Z^{2}P(100-P)}{\varepsilon^{2}}$$

Where, Z = level of confidence (1.96 for 95% confidence level), p = expected proportion = 6.6% This is the estimated prevalence of sepsis in Tanzania based on the study done in Bugando [4]. ε = margin of error = 3.3%. A minimum sample size of 239 was calculated.

**Variables:** a structured questionnaire was used to collect information from participants, including: demographic data, clinical presentation, clinical outcome, laboratory findings and duration of hospital stay. All participants received standard treatment and their laboratory results were shared with the treating paediatricians to guide their care. The participants were followed up until discharge or death. A hospital stay of > 7 days was regarded prolonged. Weight, height, and Mid Upper Arm circumference (MUAC) were measured and we used the WHO standard growth charts of 2016 to assess the nutritional status [14].

**Blood sample collection and laboratory testing:** strict aseptic technique was followed to collect blood sample from peripheral veins. Approximately 3cc of blood taken then inoculated in pediatrics blood culture bottle Bacteralert paediatric blood culture bottle (BacT/Alert PF (Organon-Teknika Corp., Durham, N.C.). We took single blood culture due to limited blood volume of the participants These were incubated at 37°C temperature for 24h after which were sub-cultured on solid agar plates; blood agar and MacConkey agar and chocolate agars (Oxoid, UK) for up 72 hours before being regarded as having no growth. For those which grew bacteria further tests were done based on microscopic characteristics, colonial characteristics, and Biochemical tests. Gram-negative organisms were identified by oxidase, Triple sugar Iron (TSI), sulphur indole and motility (SIM), urease, citrate test, VP and Methyl red test. Whereas Gram-positive organisms were catalase reaction, coagulase test, DNase test and bile esculin test Blood cultures with the following bacterial growth were regarded as contaminants *Micrococcus spp, Corynebacterium spp, Propionibacterium spp* and *Bacillus spp* [15].

**Antimicrobial susceptibility test:** this was done for first and second-line antibiotics used in the treatment of sepsis in our setting as dictated by the Tanzanian national treatment guidelines. Antimicrobials include: ampicillin and gentamycin whilst the second-line antimicrobials include: ceftriaxone, vancomycin and co-amoxiclav. The third line antimicrobial tested is meropenem. In addition, for cultures which isolated *Enterobactericiae spp* the following antibiotics were added: amikacin 30μgm, ciprofloxacin 5μgm and piperacillin-tazobactam 100μgm/10μgm. For those which isolated *Staphylococcal spp* an addition of erythromycin15μgm and clindamycin 2μgm was done to meet the Clinical Laboratory Standard Institute (CSLI) guideline. Drug concentrations used included; ampicillin 10µgm, gentamicin 10µgm ceftriaxone 30µgm, vancomycin 30µgm, co-amoxiclav 30µgm and meropenem 10µgm. Sensitivity was tested using disc diffusion method, (Kirby-Bauer) and sensitivity was based on the criteria set by the Clinical and Laboratory Standards Institute (CLSI). The results were then recorded as sensitive or resistant, depending on the zone of growth inhibition.

**Data measurement:** demographic data like age, weight height MUAC and duration of hospital stay were measured as continuous data while sex education level, susceptibility and outcome were measured as qualitative variables.

**Statistical methods:** data were analyzed using statistical package for social science (SPSS) version 20. Continuous variables were summarized using means and standard deviation and medians, and interquartile ranges. Student T-test was used to compare means of data which were normally distributed and Mann-Whitney U test was used to compare medians for skewed data. Categorical variables were summarized using frequencies and proportions. The differences in proportions were tested using Chi-square test or Fisher´s exact test where applicable. The respective 95% confidence intervals were determined and a p-value equal or less than 0.05 was considered statistically significant.

**Ethics approval:** this study was approved by MUHAS Institutional Review Board and Muhimbili National Hospital Directorate of Training and Research. Caregivers were requested to sign a written informed consent before enrollment. Children above 7 years were required to sign an assent form if they accepted to participate in the study and their caregivers concurrently signed a written consent form. Confidentiality was maintained by use of a unique study identification number for each participant. All children received the standard of care offered at MNH.

**Funding:** Muhimbili National Hospital through research grants fund.

## Results

**Figure 1:**
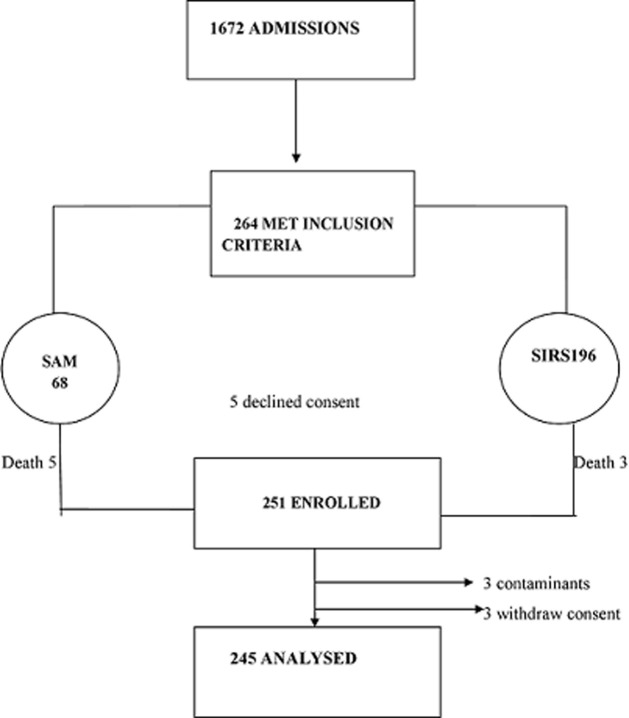
flow diagram showing recruitment of study participants

The diagram below shows the recruitment of participants, about a quarter 68 (25.8%) presented with SAM and whilst most 196 (74.2%) had SIRS. There were 8 deaths occurred before taking blood culture samples, 3 blood cultures grew *Micrococcus and Bacillus* considered to be contaminants and were discarded. Therefore only 245 samples were analyzed. As shown in Figure 1.

A total of 245 participants met the inclusion criteria with a 65.7% (161) male predominance. Majority of the participants 195 (79.6%) were less than 5 years of age with a median age of 2 years (IQR: 10 months - 4 years). Nearly half of participants (44.5%) had used antibiotics for more than 3 days prior to admission, commonly ceftriaxone 112 (45.9%). At the Emergence Medicine Department (EMD), the same antibiotic ceftriaxone was prescribed for more than half of the cases 146 (59.6%). Few children 22.9% changed to another antibiotic during hospital stay. Most of the participants were cared for by biological parents who had a median age of 34 years [IQR: (28-38]. Majority (92.7) were married, with (52.7%) having primary level of education and (45.3%) were self-employed.

### Blood culture result

**Figure 2:**
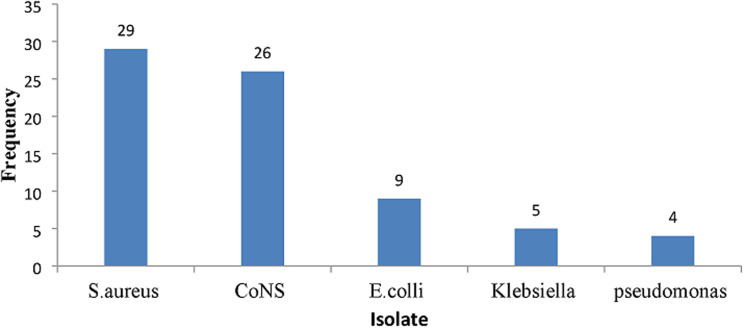
common bacterial isolates causing sepsis in children aged 2 months to 15 years admitted at Muhimbili National Hospital

The prevalence of culture-positive sepsis among the participant was (73 /245) 29.8 % (95%CI: 24.4-35.9)). Majority of positive blood cultures were participants aged between 1 and 5 years, whereby fifty one (69.9%) were male and 22 (30.1%) were female participants respectively. However, there was no significant association between age and gender with respect to blood culture results as shown in Table 1. Majority 55 (75.3%) of the bacterial isolates were Gram-positive with a predominance of *S. aureus* 29 (39.7%) and *Coagulase Negative Staphylococcus* (CoNS) 26 (35.6%); whilst the Gram-negative bacteria included: *E. coli* 9 (12.3%), *Klebsiella spp* 5 (6.8%) and *Pseudomonas aeruginosa* 4 (5.5%) as shown in Figure 2. Table 2 shows the distribution of bacterial isolates causing sepsis in different age groups whereby *S. aureus* and CoNS were more common in children aged 1 to 5 years, while *E. coli* was predominant in children above 5 years and this difference was statistically significant with a p-value 0.001.

**Table 1:** demographic characteristics and blood culture results in children with sepsis (N=245)

Variable	Culture results			
	Growth (N=73)	No growth (N=172)	Total (N^=^245)	P-value
**Child Sex**				
Male	51 (69.9)	110 (64.0)	161 (67.5)	
Female	22 (30.1)	62 (36.0)	84 (34.3)	**P=0.373**
**Age in years**				
<1	19 (26.0)	53 (30.8)	72 (29.4)	
1-5	42 (57.5)	81 (47.1)	123 (50.2)	**P=0.314**
>5	12 (16.4)	38 (22.1)	50 (20.4)	

**Table 2:** demographic characteristics and common bacterial isolates (N=245)

Variable	Bacteria Isolated						
	E. coli	S aureus	Klebsiella	CoNs	Pseudomonas	Total	P-value
**Child age (years)**							
<1	0 (0.0)	8 (27.6)	2 (40.0)	9 (34.6	0 (0.0)	19 (26.0)	
1-5	3 (33.3)	18 (62.1)	1 (20.0)	16 (61.5)	4 (100)	42 (57.5)	**P=0.002
>5	6 (66.7)	3 (10.3)	2 (40.0)	1 (3.9)	0 (0.0)	12 (16.4)	
**Sex**							
Male	7 (77.8)	21 (72.4)	3 (60.0)	17 (65.4)	3 (75.0)	51 (69.9)	
Female	2 (22.2)	8 (27.6)	2 (40.0)	9 (34.6)	1 (25.0)	22 (30.1)	*P=0.929

* Indicates that Fisher’s exact test was used to determine the association ** indicates that Fisher’s exact test was used to determine the association and the association was significant

### Antibiotics susceptibility pattern of bacteria isolates

Among the bacterial isolates, resistance to antimicrobial agents amongst both Gram-positive and Gram-negative bacteria was ranging from 5% to 100% with ampicillin (80-100%) and ceftriaxone (40-70%) as shown in Table 3. The *S. aureus* isolates showed good sensitivity of about =80% to a clindamycin, meropenem, co-amoxiclav and ciprofloxacin; but had a high resistance to ampicillin (83%), ceftriaxone (69%) erythromycin (62%) and vancomycin (44.8%). Similar findings were observed from CoNS which showed up to 96.2% of the isolates to be resistant to ampicillin (57.7%) ceftriaxone, vancomycin and erythromycin. The CoNS had a moderate susceptibility to gentamycin 61.5% ciprofloxacin (65.5%) co-amoxclav 77% and meropenem (69.2%), but were most sensitive to clindamycin (80.6%). Among the Gram-negative bacteria, *E. coli* isolates were found to be 100% sensitive to amikacin and piperacillin-tazobactam but were resistant to gentamycin (22.2%), meropenem (33.3%) co-amoxclav and ciprofloxacin (44.4%). On the other hand, *E. coli* illustrated a marked resistance to ceftriaxone (66.7%) and ampicillin (89%) respectively. Other Gram-negative bacteria such as *Klebsiella spp* also showed good response to meropenem (80%), piperacillin-tazobactam (80%) and 100% sensitivity to amikacin. Like *E. coli*, the *Klebsiella spp* also had a similar resistance pattern of (80%) to ampicillin, 60% to co-amoxclav and gentamycin and 40% to ceftriaxone and ciprofloxacin respectively. All *Pseudomonas aeruginosa* isolates were resistant to ampicillin, gentamycin and ceftriaxone but (75%) were sensitive to amikacin (Table 3).

**Table 3:** bacteria isolated and antimicrobial sensitivity pattern among children admitted at MNH

Bacteria	AMP S (%)	GEN S (%)	CEF S (%)	AM0 S (%)	VAN S (%)	CL S (%)	ME S (%)	TA S (%)	ER S (%)	CI S (%)	AM S (%)	N
S aureus	5 (17)	24 (82.8)	9 (31)	22 (75.9)	16 (55.2)	24 (82.8)	24 (82.8)	NA	11 (38)	22 (75.9)	NA	29
E. coli	1 (11)	7 (77.8)	3 (33.3)	5 (55.6)	NA	NA	6 (66.7)	9 (100)	NA	5 (55.6)	9 (100)	9
Klebsiella	1 (20%)	2 (40)	3 (60)	2 (40)	NA	NA	4 (80)	4 (80)	NA	3 (60)	5 (100)	5
CoNS	1 (3.8)	16 (61.5)	11 (42.3)	20 (77)	11 (42.3)	21 (80.6)	18 (69.2)	NA	11 (42.3)	19 (65.5)	NA	26
Pseudomonas	0	0	0	1 (25)	NA	NA	1 (25)	1 (25)	NA	1 (25)	3 (75)	4

S Sensitive | AMP Ampicillin | GEN Gentamycin | CEF Ceftriaxone | AMO Amoxiclav | CL Clindamycin | ME Meropenem | TA Piperacillin Tazobactam | ER Erythromycin | CI Ciprofloxacin| AM Amikacin | NA Not applicable

**Clinical outcomes of study participants:** the proportion of children dying from sepsis at MNH during the study period was 9.4% whilst 59.3% (132/222) of the survivors had prolonged hospital stay of >7 days There were significantly more deaths occurring in children aged between 1 and 5 years (P= 0.035), those who had prior antibiotic use (73.9%) (P = 0.003) those presenting with tachycardia of >160 beats/minute (p = 0.003) and those with leukocytosis of >11k/uL (p value=0.002). Similarly, more deaths occurred in children found to be infected with *E. coli* and CoNS bacteria however, this was not statistically significant (Table 4).

**Table 4:** socio-demographic and clinical characteristics versus outcomes of children with sepsis admitted at MNH

Variable	Clinical outcomes		
	Discharge (n=222)	Death (n=23)	P-value
**Child age (years)**			
<1	69 (31.1)	3 (13.0)	
1-5	112 (50.4)	11 (47.8)	**P= 0.039
>5	41 (18.5)	9 (39.1)	
**Gender**			
Male	147 (66.2)	14 (60.9)	
Female	75 (33.8)	9 (39.1)	P= 0.607
**Bacterial isolate**			
*S. aureus*	27 (43.6)	2 (18.2)	
*E. Coli*	6 (9.7)	3 (27.3)	*P= 0.06
*Klebsiella*	4 (6.4)	1 (9.1)	
*CoNS*	23 (37.1)	3 (27.3)	
*Pseudomonas*	2 (3.2)	2 (18.2)	
**Used antibiotics before**			
Yes	92 (41.4)	17 (73.9)	
No	130 (58.6)	6 (26.1)	**P= 0.003
**Temperature °C**			
<36.5	8 (3.6)	3 (13.0)	
36.5-38	6 (2.7)	1 (4.3)	
>39	208 (93.7)	19 (82.6)	*P=0.081
**Pulse rate b/m**			
<60	1 (0.5)	2 (8.7)	
60-120	3 (1.3)	1 (4.4)	
>120-160	121 (54.5)	9 (39.1)	
>160	97 (43.7)	11 (47.8)	**P=0.017
**Wbc**			
<4k/µl	23 (10.4)	8 (34.8)	
4k-11k/µl	97 (43.7)	5 (21.7)	
>11k/µl	102 (45.9)	10 (43.5)	P=0.002
**Change of antibiotics**			
Yes	48 (21.6)	8 (34.8)	
No	174 (78.4)	15 (65.2)	P=0.152

* Indicates that Fisher’s exact test was used to determine the association ** indicates that Fisher’s exact test was used to determine the association and the association was significant

## Discussion

The findings of this study showed a relatively low yield of culture-positive sepsis (29.8%) which may have been contributed by a 40% prior antibiotic use. Gram-positive bacteria were the predominant cause of sepsis. There was a high level of resistance against common first and second-line antimicrobial agents. Mortality was found to be higher in children aged 1-5 years presenting with prior antibiotic use, tachycardia and leucocytosis.

### Common bacteria causing sepsis in children at Muhimbili National Hospital MNH

In this study, the prevalence of culture-positive bacterial sepsis was 29.8%. This finding is higher than what has been reported in Iran 9.1%, Ethiopia 18.2%, Kenya 6.4% and previous studies done in Tanzania and Zanzibar which have positive culture results in 6.6%-14% of the children. However, it is lower than blood culture yields reported in Nigeria 35% and Zimbabwe 37.1% respectively [16-26]. These discrepancies can be due to: study design, difference in blood culture system, seasonal variation, varying levels of infection control methods and nature of participants selected.

The common bacteria isolated were *S. aureus, E. coli, Klebsiella spp*, and *Pseudomonas aeruginosa*. This was comparable to bacteria isolated in studies done in developed countries, although other bacteria like *Acinobacter, Neissseria meningitidis and Streptococcal pneumonia* predominated [9,8,10,27,28]. Similarly, in a meta-analysis done in Africa in 2011, similar bacteria were isolated but there was predominance of *S. pneumoniae* isolates in children with *sepsis* [6]. This difference in bacterial isolates can be due epidemiological and geographical distribution of bacteria and seasonal variation. *Streptococcal pneumonia* were the leading isolates found in sub-Saharan countries in 2011 [6], however, there has been a decline in *S. pneumonia* related sepsis since the introduction of the pneumococcal conjugate vaccine (PCV) globally (28) and in Tanzania since 2012 [29,30]. The low prevalence of *Streptococcal*isolates could also be contributed by the prior use of antibiotics reported among 44.4% of the participants. The most commonly used antibiotics in our setting are the penicillin group which could either have been self-medicated or prescribed at lower level health facilities.

In this study CoNS were the second most common bacteria isolated contrary to studies in developed countries were no such isolates have been reported. Early in the 1970s CoNS were regarded as contaminants but several studies has reported an increased incidence of CoNS in children with sepsis [13,21,23-25,31,32]. In Ethiopia, CoNS isolation was higher amongst 26.1% to 43.3% of their participants; whereas a similarly higher prevalence was observed in Tanzania as it was the second most common isolate between 2010 to 2012 [21,31]. CoNS occur commonly in children who are immune-compromised and those on advanced medical care such as mechanical ventilation and, central line catheterization. The isolation of CoNS among such patients reflects CoNS as a true pathogen and not a contaminant. In this study, some children underwent central line catheterization and about 27.8% had severe acute malnutrition which is state of immune suppression.

Notably, in this study there was a predominance of Gram-positive bacteria contrary to studies done in India, Kenya, Malawi and South Africa where Gram-negative bacteria predominated. In Tanzania, at MNH there has been a shift from Gram-negative to Gram-positive bacteria since 2007 [17,20,21,23, 28,32-34]. This can possibly be due to seasonal variation in the patterns of organism causing sepsis or differences in the study population because previous studies involved neonates who are normally infected by Gram-negative bacteria.

### Antibiotic susceptibility pattern for bacterial isolates

There was an increased resistance to the commonly used antibiotics which are: ampicillin, ceftriaxone, vancomycin, erythromycin, co-amoxiclav, gentamycin and ciprofloxacin. This was similar to what has been observed in several studies in developed and developing countries [4,6,9,17,20,34-37]. However, there was less resistance to gentamycin and co-amoxiclav compared to a previous study done at Bugando in Western Tanzania [4]. The same resistance pattern has also been reported among neonates with sepsis admitted at MNH [16,17], implying that antibiotic resistance is a burden in all age groups.

There was notably an increase in resistance to ceftriaxone up to 50% compared to previous studies which reported up to 40% [30,32]. This is possibly due to overprescription of the drug since more than 50% of the participants used ceftriaxone prior to admission. This trend of increased resistance was also noted with meropenem whereby previous studies had reported a 100% sensitivity [20,22] and the current study showed a sensitivity of 25% to 82.8% only. This is reflected by the increased prescription of meropenem because most children admitted at MNH have already used cephalosporins.

From this study both Gram-positive and negative bacteria showed good sensitivity to ciprofloxacin. Previously ciprofloxacin was not prescribed in children due to fear of long-term arthropathy based on findings from animal studies. However studies done in children showed it is reversible arthropathy [38]. It has been recommended by the WHO for treatment of septicemia not responding to other medications [39,40]. Therefore, from our findings, ciprofloxacin can be recommended as an alternative 2nd line antibiotic. *Staphylococcal spp* isolated showed good sensitivity to clindamycin which is rarely prescribed at MNH. Therefore it can be reserved for severe *Staphylococcal* infections which do not respond to the conventional antimicrobials. On the contrary, *Pseudomonas aeruginosa* isolates showed a high resistance to most antibiotics used at MNH because it is a common cause of hospital acquired infections in patients already exposed to several antimicrobials. Several studies have also reported an increase in antimicrobial resistance in children with hospital acquired infections [18,20,41,42], which poses a challenge in the treatment of sepsis.

### Clinical outcome of children with sepsis admitted at MNH

The case fatality rate (CFR) from sepsis in this study was 9.4% which is lower than the rate documented in India 32.7% (33). It is equally lower than previous findings in Tanzania, which showed a case fatality rate of 14.2% in 2017 and 13.1% in 2018 respectively [43,44]. This shows significant decrease in childhood mortality due to sepsis at MNH from 14.2% in 2017 to 9.4% in 2020, which may be due to a significant improvement in healthcare services. On the other hand, the CFR rate reported in this study is higher than the average national case fatality rate of 6.7% as reported by the Tanzania Demographic and Health Survey of 2015-16 [44], Similarly, the CFR was higher when compared to developed countries with CFR rate of 5.6% [7], This can reflect the level of care between developed and developing countries, whereby all children with sepsis were admitted in intensive care unit (ICU) in developed countries compared to sub-Saharan countries.

It was observed that more deaths occurred in the age between 1 to 5 years, those who had used antibiotics prior to admission, those presenting with tachycardia and leucocytosis. This is possibly they had been sick for long time. More than half of survivors had a prolonged hospital stay of > 7days contrary to reports from other studies which showed a hospital stay of 4 to 6 days [33,43]. This can be due to the severity of sepsis at the time of admission leading to a delayed discharge.

### Strengths and limitations

The study employed the SIRS criteria for diagnosis of sepsis which is more accurate compared to the use of any fever as proxy to sepsis. Children with severe acute malnutrition were also included in this study as a representative sub-population at high risk of sepsis and sepsis-related complications, hence, this enhanced culture yield as this group may be missed using the SIRS criteria. This was a hospital-based single-centre study hence the results cannot be generalized. At the same time, up to forty percent of the children referred to MNH were already on antibiotics; which could have resulted into a false culture-negative sepsis and/or a low yield for some of the bacterial isolates. We miss oxacillin disc to test for MRSA and MIC was also not done.

## Conclusion

Bacterial sepsis contributes to a high mortality of 9.4% and a prolonged hospital stay of more than 7 days among 59.3% of the participants. Gram-positive bacteria were found to be predominant cause of sepsis; both Gram-positive and Gram-negative bacteria had a high resistance to first and second-line antimicrobials including: ampicillin, gentamycin, and ceftriaxone. We recommend a revision of the standard treatment for sepsis in our guidelines to reflect the current antimicrobial sensitivity patterns whilst continuing with antimicrobial surveillance studies. Antimicrobials with a good sensitivity pattern such as clindamycin and ciprofloxacin should be reserved for severe infections which do not respond to conventional antibiotics. Meanwhile, Children with sepsis aged between 1 and 5 years, presenting with prior antibiotics use, having tachycardia and leukocytosis should be considered critical and receive intensive care to reduce their risk of dying.

### What is known about this topic

- Sepsis is the leading cause of mortality;
- There is an increase in antimicrobial resistance to first-line antibiotics used for the treatment of sepsis.

### What this study adds

- There is still high mortality due to sepsis;
- There is an increase in resistance to 2nd and 3rd line antibiotics used in sepsis treatment.
